# Monthly Summary

**Published:** 1880-02

**Authors:** 


### MONTHLY SUMMARY.

---

*Treatment of Fistulæ and Scars of the Cheek.*—On this subject Mr. Edward Bellamy, F. R. C. S., writes that it is, in the first place, all-important to find out exactly the course taken by the fistula or fistulæ—a matter of considerable difficulty sometimes; and the following classification may have its value in diagnosis: 1.—Those opening into the cheek, with a track above the level of the buccal or labial mucous membrane, and which usually discharge saliva only. 2.—Those whose track lies below this level, and which discharge pus and mucco purulent fluid and no saliva. 3.—A complication of both forms, and which discharge both pus and saliva. With regard to the accurate detection of their course, an ordinary probe frequently gives merely a general idea of the direction, without passing into the offsets. I have always found that a fine filiform bougie, or better still, a fine india-rubber French bougie, is more useful than anything else. After having determined the course, irritating cause, and condition of the fistula, in order to avoid further scar, the dead bone, if there be any, is to be removed by delicate but strong forceps or gouge, and afterward the track should be washed out with a very strong solution of sulphuric acid, which has the effect of completely destroying the fistulous track; or by the introduction of minute crystals of nitrate of silver, until the granulations appear at the orifice, gentle pressure being maintained. A cicatrix, however carefully the treatment be carried out, is sure to remain, unsightly always and often troublesome, appearing as a "pucker" or adhesion to the underlying bone: and with regard to its treatment, I venture to state, from my own experience, that two methods are open to the surgeon, dependent on the extent or strength of these adhesions. The first consists in introducing a fine blunt-pointed tenotome through the tissue of the cicatrix—laminating it, as it were—taking great care to leave it in free communication with the integument adjacent to it; next, to introduce between the split surfaces a thin strip of sheet-lead, which should be kept in, to prevent the adhesion of the surfaces divided by the tenotome. After a few days, the superficial lamina of the cicatrix may be subjected to gentle movement over the lower lamina, which the patient may conduct himself; this prevents adhesion, and renders the tissue pliant and assimilative. This may be termed the "passive movement" of the cicatrix.—*London Lancet.*

---

*Effects of Arsenic on the Body.*—An extensive series of experiments on pigs, rabbits and fowls by C. Gies, published in the *Lancet*, gives some very interesting results. The quantity given was exceedingly minute, being only for rabbits 0.0005 of a gramme, (120th of a grain) and to the pigs and fowls not greatly more; the dose continued for four months. The following changes occurred: The weight increased; the subcutaneous fat was augmented; the bones developed considerably in length and girth, and they presented that peculiarity that *wherever in the normal state spongy tissue exists it was replaced by compact bone.* The bones of the corpus and tarsus were thus converted into *solid bony masses.* Moreover a compact layer of bone was found immediately beneath the epiphysical cartilages of the long bone; a condition identical with animals fed with phosphorous in their food. This change was apparent when the total of doses amounted only to 0.02 of a gramme of arsenic. Other grave changes, viz:—enlargement of the spleen, heart, liver and kidneys were noticed. A very peculiar fact was noticed, that of similar changes in the bones of animals kept in the same cages. This was supposed to be due to the inhalation by them of particles of arsenic with which the air was loaded. [Is this a possible thing? Recalling the minuteness of the dose, and the manner of the alleged poisoning, is it possible for this injurious elimination to occur? It beats Dr. Flagg's frogs.—Eds.] The young of such animals were always still-born, and were marked by the same hypertrophy of spleen and incipient changes in the bones.

---

England cannot afford a microscopical journal. The one lately published in that country is dead.

*Death from a Tooth in the Trachea.*—In New York lately, a hospital patient, upon whom an operation affecting the jaw was to be performed, suddenly vomited while a tooth was being extracted. The tooth slipped from the forceps and disappeared. The patient coughed considerably on the next day, and also on the day following, when Dr. Legerts, the house-surgeon, made the diagnosis that the tooth had entered the air-passages, had lodged in the left bronchus, completely obstructing it.

Evidence of pleurisy followed, and subsequently evidence of lung consolidation. The entrance of air into the left lung was completely prevented until abscesses had formed, when air could be heard passing the tooth. No operation was performed for the removal of the foreign body. The case terminated fatally. At autopsy the tooth and abscesses were found as diagnosticated.

---

*Saliva and the Digestion of Starch.*—Dr. R. M. Smith, in a lecture on experimental physiology, at the University of Pennsylvania, showed that the gastric juice only suspended the action of saliva in changing starch to sugar, the action being resumed when the acidity is neutralized by the intestinal juices. He showed also that while caustic alkalies destroy the catalytic action of saliva, the weaker alkalies only suspend it. This proves the rationality of giving these alkalies in acidity of the stomach or mouth. It gives a better chance for the digestion of amylaceous foods.—*Med. and Surg. Reporter.*

---

*Relative Success of Different Treatment of Typhoid Fever.*—Dr. Samuel Peters, in *N. Y. Medical Record* in summing up the relative success of different methods of treatment of typhoid fever, states that under the old energetic treatment of 1843-64 the mortality was 27 per cent.; while under the antipyretic treatment it is reduced to 15 per cent. A number of authorities are quoted to the effect that under the cold water treatment the mortality was over 20 per cent.; and under customing or food treatment only about 4½ per cent. The best record is from the lime-water and milk treatment, wherein some reports all recovered.

---

*A Question.*—The bill of an M. D. was presented, without items. The patient requested to have it itemized; whereupon the doctor thought it strange the party in question could trust his life in his (the doctor's) hands, but could not trust his money matters.

---

*Arsenic in Bismuth.*—According to a French authority, Bismuth always contains a proportion of arsenic, and its continued use is therefore pernicious.

